# The Effect of Arterial pH on Oxygenation Persists Even in Infants Treated with Inhaled Nitric Oxide

**DOI:** 10.1155/2011/189205

**Published:** 2011-07-03

**Authors:** Aimee M. Barton, M. Kabir Abubakar, Jennifer Berg, Martin Keszler

**Affiliations:** ^1^Department of Pediatrics, Georgetown University Hospital, 3800 Reservoir Road NW, M3400, Washington, DC 20007, USA; ^2^Department of Pediatrics, Women and Infants Hospital, 101 Dudley Street, Providence, RI 02905, USA

## Abstract

*Objective*. To validate the empiric observation that pH has an important effect on oxygenation in infants receiving iNO. *Study Design*. Demographics, ventilator settings, arterial blood gases (ABG), and interventions for up to 96 hours of life were extracted from the charts of 51 infants receiving iNO. Need for ECMO and survival to discharge were noted. Mean blood pressure (MBP) and mean airway pressure (MAP) were recorded. The arterial/alveolar (a/A) ratio was used as the primary outcome. Analysis was by simple linear regression and multiple linear regression analyses and Fisher's exact test. pH responsiveness was arbitrarily defined as a correlation coefficient (CC) of >0.40 with *P* < 0.05. *Results*. Mean gestational age was 38.8 weeks and mean birth weight was 3300 g. All patients had clinical diagnosis of PPHN. Clear responsiveness to pH was found in 31/51 infants. MAP and MBP did not correlate with a/A ratio. Three responders had a critical pH > 7.55. Of 11 patients requiring ECMO, only 3 exhibited responsiveness at any time in their course. Three responders required ECMO. *Conclusion*. This small study suggests that failure or inability to optimize pH may account for observed unresponsiveness to iNO. Maintaining a pH > 7.5 using hyperventilation is not recommended.

## 1. Introduction

Hypoxemic respiratory failure continues to be a significant source of morbidity and mortality for term and near-term infants and is the most common reason for neonatal extracorporeal membrane oxygenation (ECMO) referral. Hypoxemic respiratory failure is commonly associated with persistent pulmonary hypertension of the newborn (PPHN), a syndrome characterized by failure to achieve or maintain the normal decrease in pulmonary vascular resistance (PVR) that occurs after birth [1]. Increased PVR leads to pulmonary hypertension, right ventricular dilatation, tricuspid insufficiency, myocardial dysfunction, and extrapulmonary right-to-left shunting, which leads to severe hypoxemia that is often unresponsive to conventional therapy [2]. ECMO remains a treatment of last resort, reserved for infants who fail to respond to such therapies. 

In the last decade and a half, multiple randomized controlled trials have shown that iNO improves oxygenation and decreases the need for ECMO in term and near-term infants with hypoxemic respiratory failure [3–8]. However, up to 40% of infants treated with iNO in these pivotal trials required ECMO. Response rate to iNO is inversely related to the severity of pulmonary disease and is facilitated by optimizing lung aeration by employing interventions such as exogenous surfactant and high-frequency ventilation. Despite these measures, a significant proportion of infants do not respond to iNO.

In our NICU at Georgetown University Hospital, iNO has been in routine use for infants with hypoxic respiratory failure who do not improve with high-frequency ventilation, oxygen, inotropic support, and surfactant (when appropriate, as in cases of RDS in late preterm infants or meconium aspiration syndrome) since the late 1990s. We have made the empirical observation that infants receiving iNO are more likely to respond if their arterial pH is within high normal range of 7.40–7.45 than if it is in the low normal or acidotic range (< 7.35). Infants who had initially failed to respond to iNO often demonstrated responsiveness when we subsequently achieved a higher arterial pH.

Though Heidersbach et al. demonstrated in an animal model that a systemic arterial pH > 7.40 augmented iNO-induced pulmonary vasodilation, extensive review of the literature failed to reveal any clinical observation of this phenomenon. The objective of this study was to explore the relationship between arterial pH and oxygenation in near-term infants with severe hypoxemic respiratory failure who were being treated with iNO.

## 2. Study Design

After obtaining HIPAA waiver, all term and near-term infants who received iNO treatment for PPHN in the Georgetown University Hospital NICU during a 5-year period were identified from our neonatal information system (NIS) database. The medical records were obtained, and each was assigned a sequential number not related to the medical record number for organizational purposes.

Available records were reviewed, and the following information was recorded by the principal investigator (AMB): gestational age, birth weight, gender, ethnicity, maternal age, age in hours at time of presentation, primary diagnosis, echocardiographic evidence of PPHN (elevated right ventricular pressures with or without right to left shunting), inborn/outborn status, use of dopamine, dobutamine, epinephrine, or hydrocortisone, use of antibiotics, and outcome (death, ECMO, or recovery without ECMO). The information was recorded into a data collection form and was organized by the assigned number.

Additional data collected for each infant included a baseline arterial blood gas (ABG), defined as the ABG prior to initiation of iNO; baseline ventilation mode and settings just prior to initiation of iNO therapy; age in hours at initiation of therapy; post-iNO ABG (first gas after initiation of therapy); all available ABG results for the first 96 hours on iNO (or until iNO was discontinued for recovery, ECMO or death); mean blood pressure (MBP) at the time of the blood gas determination; mean airway pressure (MAP) at the time of blood gas determination; ventilation changes; “pH interventions” (defined as sodium bicarbonate, THAM, or fluid bolus); additional interventions such as surfactant administration.

In our NICU, metabolic acidosis in infants with PPHN is corrected with fluid boluses and sodium bicarbonate. Fluid boluses are also used to maintain blood pressure if other signs of hypovolemia (tachycardia, poor perfusion) are present. THAM is used only in cases of metabolic acidosis with poor ventilation (increased PaCO_2_). It is not our practice to use sodium bicarbonate or THAM to induce alkalosis. Hydrocortisone is given in our unit for hypotension refractory to volume expansion and maximum dopamine and dobutamine support. Hourly MAP and MBP are available on all of our PPHN patients, and scheduled blood gas determinations are usually taken on the hour. The MAP and MBP closest to the time of all blood gas determinations were recorded. Frequency of ABG measurements is based on clinical status of the infant, ranging from hourly (or more frequently) in the early, more critical period, to every two to four hours once the infant has stabilized. Our practice with respect to target PaCO_2_ has evolved over time, shifting away from aggressive hyperventilation to more recently maintaining PaCO_2_ levels within a normal to mildly alkalotic range (30–45 torr). During the earlier period of the study, there was some practice style variation leading to moderate hyperventilation in some infants.

The a/A ratio [PaO_2_/(760–47) × (FiO_2_/100)-PCO_2_], rather than PaO_2_, was used to assess oxygenation in order to account for the effect of changes in PaCO_2_ and FiO_2_. We elected not to use the oxygenation index (MAP × FiO_2_ × 100/PaO_2_), because mean airway pressure does not correlate well with oxygenation when the cause of hypoxemia is extrapulmonary, rather than intrapulmonary shunting.

To protect privacy of patient information, the principal investigator kept the assignment log in a password-protected file. The study was approved by the Georgetown University Institutional Review Board, which granted waiver of HIPAA and informed consent requirements.

## 3. Statistical Methods

First we performed univariate analyses using simple linear regression to determine the correlation coefficient (CC) for pH, MBP, MAP, and time since start of iNO versus a/A ratio. Responsiveness to pH in each infant was arbitrarily defined as a positive correlation coefficient of >0.40 with a *P* value of < 0.05. Because pH sensitivity wanes as vasoconstriction subsides, analysis was limited to the period of responsiveness, if present. This period was determined by consensus of the investigators using visual examination of the pH and PaO_2_ data. Multiple linear regression analysis was then performed to determine possible independent effect of each variable (pH, MBP, MAP, and time) on responsiveness. Fisher's exact test was used to compare the need for ECMO in pH responders versus nonresponders. Each patient's data plotted in a pH versus PaO_2_ graph were visually examined to determine the “critical pH” at which oxygenation responsiveness was achieved. For patients with a clear inflection point to their a/A ratio versus pH graph, the critical pH was determined by the value of pH at the inflection point. Nineteen of thirty-one pH-responsive infants had such an inflection point. In the remaining infants who had a fairly linear relationship between pH and a/A ratio, we arbitrarily chose the pH at which PaO_2_ exceeded 100 mmHg with FiO_2_ of 1.0 as the “critical pH.”

## 4. Results

Fifty-nine potential study patients were identified. Two records could not be located and of the remaining fifty-seven infants two were excluded due to prematurity, one because of unavailability of arterial blood gases (no arterial line), one due to congenital heart disease recognized only after being placed on iNO and two because of incomplete data. In total, fifty-one records were retained for analysis.

Table 1 shows the demographics of the study patients. There was no statistical difference in mean birth weight, mean gestational age, primary diagnosis, or mean age at iNO initiation between pH responders and non-responders. As expected, there were more males than females in each group, with a higher proportion of males falling into the “pH nonresponsive” group.

Ninety-two percent of the infants were outborn. All infants received antibiotics for treatment of possible sepsis and dobutamine and dopamine (at maximum dosage of 20 mck/kg/min) for blood pressure support. Thirty-two of fifty-one patients (63%) received hydrocortisone for blood pressure support. Forty-seven of 51 infants (92%) had elevated right cardiac pressures, suggesting pulmonary hypertension. Seventy-three percent of the infants were on high-frequency ventilation (HFV) at the initiation of iNO, including sixty-nine percent of pH responders and seventy-seven percent of nonresponders. One infant in each group was treated with high-frequency oscillatory ventilation (HFOV), the remaining thirty-five with high-frequency jet ventilation (HFJV). All infants were started at a dosage of 20 ppm iNO.

Thirty-one of fifty-one infants were classified as pH responders (61%) using the criteria listed in the statistical methods section. Among responders, the critical pH ranged from 7.3 to 7.6 with twenty of thirty-one infants demonstrating critical pH of < 7.5. Only three infants had a critical pH > 7.55.

**Figure 1:**
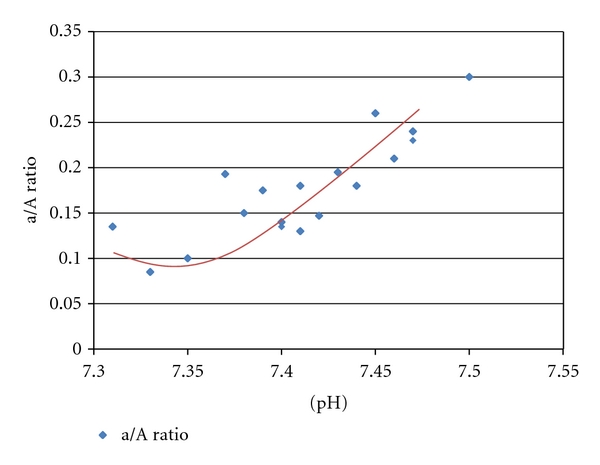
Linear response of a/A ratio to arterial pH in an individual patient. Correlation coefficient = 0.836,  *P* < 0.01.

**Figure 2:**
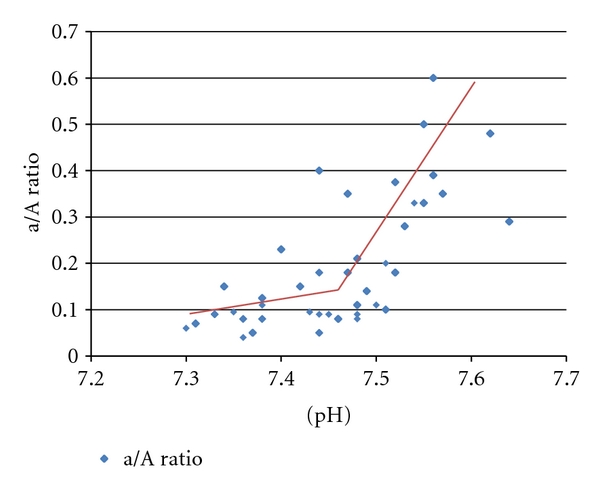
An extreme example of a threshold response of a/A ratio to arterial pH in an individual patient. Correlation coefficient = 0.614, *P* < 0.01. In this instance simple linear regression is not the optimal statistical approach, as the relationship is clearly not linear.

As described in the Methods section, two patterns of pH responsiveness for individual patients were observed: a fairly linear relationship between pH and a/A ratio (Figure 1), or a clear threshold-response of a/A to pH (Figure 2). The effect of pH on oxygenation waned over time as oxygenation improved and the infant transitioned from predominantly PPHN pathophysiology to a phase where parenchymal lung disease predominates. The data presented in the Figures and subsequent analyses represent the period of responsiveness for those patients who exhibited the pH response.

**Figure 3:**
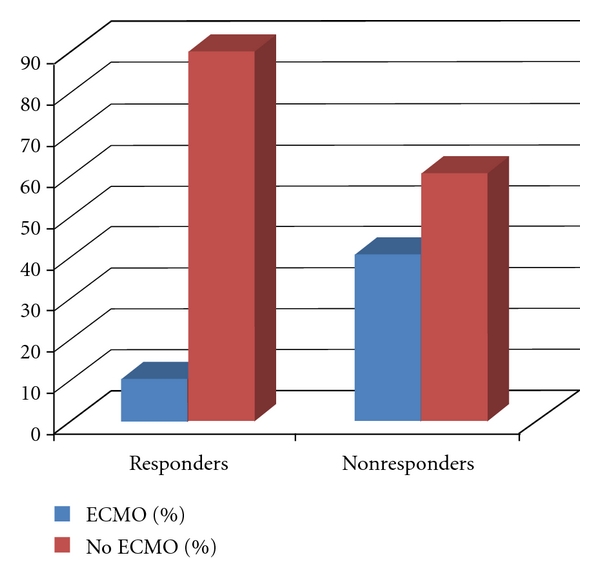
Need for ECMO in responders versus nonresponders.

Three infants who were pH responders early in their course ultimately required ECMO. All three patients had critical arterial pH > 7.5. Eight of the 20 (40%) “pH non-responsive” infants required ECMO: three infants were cannulated within 12 hours of life, one within 3 hours of arriving at our center. Two of the patients classified as pH non-responsive never achieved a pH ≥ 7.3, and one had a best pH of 7.37. Four of the remaining five infants did attain a pH ≥ 7.5, but without a sustained response in PaO_2_. Patients who were pH responsive were much less likely to need ECMO (10% versus 40%, *P* = 0.012, OR 0.16, 95% CI 0.027–0.84, Figure 3). Only one study patient died. This infant was a nonresponder and died before ECMO could be instituted with a diagnosis of sepsis and pneumothorax.

By multiple logistic regression, pH but not mean blood pressure was independently correlated with oxygenation. In pH-responsive infants, mean blood pressure showed no correlation with a/A ratio, while there was a weak (nonsignificant) correlation between MBP and oxygenation in pH non-responsive infants. Contrary to the usual positive correlation of MAP and oxygenation, MAP showed a negative correlation with a/A ratio suggesting that poor oxygenation prompted increases in MAP, to which the infants did not respond, because the etiology of the hypoxemia was extrapulmonary, rather than intrapulmonary shunting. There was no significant association between any of the other therapeutic interventions we examined and a/A ratio.

Because the critical pH varied greatly between patients, the correlation was much higher for individual patients (range 0.427 to 0.938, *P* < 0.0001 to  0.04) than for all pH responders as a group, although the group as a whole did show a highly statistically significant response to pH (CC = 0.302, *P* < 0.0001).

## 5. Discussion

Achieving optimal aeration of the lungs, adequate sedation, and blood pressure support are recognized as important factors in facilitating responsiveness to iNO. The role of alkalosis has long been appreciated in the treatment of PPHN, and, despite only limited evidence of short-term safety (with no long-term follow-up) [9], it was for many years the mainstay of treatment for this condition. A 1999 study by Heidersbach and colleagues demonstrated dose-dependent pulmonary vasodilation in a lamb model in response to oxygen, alkalosis, and iNO. They proceeded to demonstrate that when the therapies were used in combination, a systemic arterial pH > 7.40 augmented iNO-induced pulmonary vasodilation [10]. The present review was undertaken to examine the validity of the empiric observation that many babies with PPHN continue to exhibit pH-responsiveness when receiving treatment with iNO. Using the previously described criteria (positive correlation coefficient of > 0.40 with a *P* value of <0.05, for a/A ratio versus pH), pH responsiveness was found in 61% of the infants studied—however, some degree of responsiveness was seen in many other infants who did not meet the strict criteria for pH response. There were five infants who always had a pH > 7.40 and maintained good oxygenation once started on iNO. By our definition, they were classified as non-responders, though their pH sensitivity was not, in fact tested. Responsiveness to pH, when present, typically manifested itself within the first few hours of iNO therapy.

As expected, those who were pH responsive were much less likely to need ECMO. The rate of 40% need for ECMO in non-responders is consistent with the pivotal iNO trials, while the rate of 10% in responders is much lower. The overall use of ECMO in our institution for infants receiving iNO during this period was 24%.

We suspect that three of the infants cannulated for ECMO in the non-responsive group may not have achieved a sufficient pH to determine if they may have been pH responsive. They were cannulated within 12 hours of life—one within three hours of arrival to our institution. It is unknown whether better pH simply could not be achieved or whether higher pH was not attempted in these patients.

Induced systemic hypertension is often used to overcome the high pulmonary pressures encountered in PPHN. While we still strive to maintain adequate blood pressures in infants with PPHN, we found that attaining higher than normal blood pressure was not significantly correlated with response to therapy in our pH-responsive infants. It was, however, weakly correlated with response to therapy in pH non-responsive infants, suggesting that the MBP effect may be simply masked by the more dramatic pH effect.

Most infants with pH-responsiveness had a critical pH in the range of 7.4–7.5. All three pH responsive infants who eventually required ECMO had a “critical pH” greater than 7.5. Indeed, in some instances, it was a conscious decision to forgo such degree of alkalosis and, when adequate oxygenation could not be maintained with more normal pH, ECMO therapy was initiated. Our data suggest that the likelihood of sustained benefit is low if iNO responsiveness cannot be achieved at modest degrees of alkalosis. It has been observed that production of hypocapnia in infants with PPHN counteracts hypoxic vasoconstriction, thereby allowing increased blood flow [11]. This effect appears to be more related to the change in arterial pH than hypocapnia [11], and therefore, overventilating infants for this purpose is not recommended in light of potential long-term lung damage from such an action. Because of this and the possibility of serious adverse effects of hypocapnia on cerebral blood flow [12, 13], we strongly believe that infants should not be maintained with pH > 7.5 or PaCO2 < 30. In our institution, any infant that continues to require pH greater than 7.5 to achieve iNO responsiveness is now considered a candidate for ECMO, even in the absence of other indications such as hemodynamic instability, and so forth. 

In analyzing our data, we recognized that pH and PaO_2_ values much higher than desired occurred with some frequency. These values were typically seen in infants on high-frequency ventilation where even a modest increase in tidal volume can result in a large increase in minute ventilation and thus hypocapnia, because of the geometric relationship between tidal volume and minute ventilation (minute ventilation is proportional to tidal volume [2]). Furthermore, though right-to-left shunting affects oxygenation more dramatically than CO_2_ removal, decreased pulmonary blood flow will reduce CO_2_ removal proportionally. When a “critical pH” is reached and the shunt is reversed resulting in a substantial increase in pulmonary blood flow, the pCO_2_ will often drop more than desired. For this reason, we now monitor PCO_2_ transcutaneously in all infants with PPHN, especially when on HFV. Excessively high PaO2 also commonly occurs in these infants when the extrapulmonary right-to-left shunt is reversed, and may remain well above target values for some time as the FiO_2_ is weaned, sometimes too cautiously. Thus, it is clear that the effects of interventions aimed at optimizing ventilation must be carefully monitored in order to avoid excessive degrees of alkalosis, hypocapnia, and hyperoxia. 

This study is limited by its retrospective nature, lack of long-term followup, and relatively small numbers. Some of the infants did not experience a wide enough range of pH to determine the effect of pH on their response to iNO. The relationship we demonstrated is merely an association and may not be causative, though there clearly is a plausible pathophysiologic mechanism for the observed relationship, and similar relationship was observed in the laboratory [14]. It could be argued that the observed association between higher pH and improved oxygenation reflects improved ventilation-perfusion ratio as a result of the selective vasodilatation of the well-ventilated portions of the lung. However, there were many instances where, after an initial improvement in oxygenation at a higher pH, the ventilator settings were reduced and with rising PaCO_2_ and falling pH, there was a reversion to hypoxemia, even while the iNO therapy continued. This leads us to believe that the effect goes beyond the direct influence of iNO on the effectiveness of ventilation. Although sodium bicarbonate infusions were occasionally used to correct metabolic acidosis, the major contributor to pH changes was reduction in PCO_2_. It is possible, therefore, that some of the observed “pH responsiveness” may also be due to improved delivery of the iNO as a result of increased ventilator support and improved lung aeration.

As in all retrospective analyses, certain arguably arbitrary decisions and judgments had to be made. It is well known by most clinicians that infants with PPHN eventually lose their responsiveness to alkalosis. This is not an abrupt change, but rather a gradual transition from a phase during which pulmonary hypertension is the primary cause of the oxygenation defect to a “transitional phase” during which ventilator-induced lung injury plays an increasingly prominent role and pulmonary hypertension subsides [15]. This phenomenon was clearly seen in many of our infants who early in their course had a well-defined critical pH, but over time this responsiveness disappeared. We had to make the most objective decision we could as to when this transition occurred and when to truncate the data analysis. While we recognize this to be a potential limitation of this review, we do not believe this is a serious problem, because, while a flawed judgment in this process could change a pH responsive patient into a non-responder, the opposite is not true; if there were not a finite initial period of pH responsiveness with a sufficient number of data-points to reach statistical significance, eliminating later values would not create a significant correlation coefficient. Therefore, the risk, if any, is to underestimate the number of responders, an issue already addressed elsewhere. Our definition of “critical pH” in infants with a relatively linear relationship as the pH at which PaO_2_ was > 100 was quite arbitrary. The purpose was to define a level at which most, if not all, clinicians would agree that oxygenation is more than adequate. Despite this rather “conservative” definition, the “critical pH” was below 7.5 in a large majority of the infants, supporting the concept that marked alkalosis is not only undesirable [14], but usually also unnecessary. 

Despite the above limitations, based on our data, we propose that carefully performed optimization of pH in the high normal range should be considered as a potentially useful adjunct to iNO therapy and should be added to the list of measures that might be taken to optimize an infant's chance to achieve an optimal response to iNO. We do not have developmental follow-up data to inform whether the mild to moderate alkalosis that helped to avoid the need for ECMO was associated with adverse neurodevelopmental outcomes. Therefore, it must be recognized that a definitive assessment of the risks and benefits of mild alkalosis is not possible without an adequately powered prospective randomized trial with long-term followup aimed at measuring both neurodevelopmental and pulmonary outcomes.

## Figures and Tables

**Table 1:** Demographic data for study patients according to pH responsive status.

	Responders (*n* = 31); (60.7%)	Non-responders (*n* = 20) (39.2%)
Mean birth weight	3343 gm (2040–5178)	3257 gm (2670–4200)
Mean gestational age	39 wk (36.14–41.86)	38.45 wk (35.14–41.71)
Males : females	17 : 12	16 : 6

Primary diagnosis		
Meconium aspiration syndrome (MAS)	12 (39%)	13 (65%)
Congenital diaphragmatic hernia (CDH)	1 (3%)	2 (10%)
Pneumonia	4 (13%)	1 (5%)
Asphyxia	1 (3%)	2 (10%)
Blood aspiration	2 (6%)	0
Sepsis	5 (16%)	4 (20%)
Cardiomyopathy	1 (3%)	0
Respiratory distress syndrome (RDS)	3 (10%)	0
Mean age at iNO initiation	23.2 hours	25.1 hours

ECMO	3 (10%)	8 (40%)
No ECMO	28 (90%)	12 (60%)
Survival to discharge	31 (100%)	19 (95%)
